# Games to support teaching clinical reasoning in health professions education: a scoping review

**DOI:** 10.1080/10872981.2024.2316971

**Published:** 2024-02-23

**Authors:** Gilbert Koelewijn, Marije P. Hennus, Helianthe S. M. Kort, Joost Frenkel, Thijs van Houwelingen

**Affiliations:** a Research Group Technology for Healthcare Innovations, Research Centre for Healthy and Sustainable Living, University of Applied Sciences Utrecht, Utrecht, the Netherlands; b Department of Pediatrics, Wilhelmina Children’s Hospital/University Medical Center Utrecht, Utrecht, the Netherlands; c Center for Research and Development of Health Professions Education, University Medical Center Utrecht, Utrecht, the Netherlands; d Building Healthy Environments for Future Users Group, Eindhoven University of Technology, Eindhoven, the Netherlands

**Keywords:** Clinical reasoning, serious games, reflective practice, problem-solving, experimental learning, medical education, critical thinking

## Abstract

**Introduction:**

Given the complexity of teaching clinical reasoning to (future) healthcare professionals, the utilization of serious games has become popular for supporting clinical reasoning education. This scoping review outlines games designed to support teaching clinical reasoning in health professions education, with a specific emphasis on their alignment with the 8-step clinical reasoning cycle and the reflective practice framework, fundamental for effective learning.

**Methods:**

A scoping review using systematic searches across seven databases (PubMed, CINAHL, ERIC, PsycINFO, Scopus, Web of Science, and Embase) was conducted. Game characteristics, technical requirements, and incorporation of clinical reasoning cycle steps were analyzed. Additional game information was obtained from the authors.

**Results:**

Nineteen unique games emerged, primarily simulation and escape room genres. Most games incorporated the following clinical reasoning steps: patient consideration (step 1), cue collection (step 2), intervention (step 6), and outcome evaluation (step 7). Processing information (step 3) and understanding the patient’s problem (step 4) were less prevalent, while goal setting (step 5) and reflection (step 8) were least integrated.

**Conclusion:**

All serious games reviewed show potential for improving clinical reasoning skills, but thoughtful alignment with learning objectives and contextual factors is vital. While this study aids health professions educators in understanding how games may support teaching of clinical reasoning, further research is needed to optimize their effective use in education. Notably, most games lack explicit incorporation of all clinical reasoning cycle steps, especially reflection, limiting its role in reflective practice. Hence, we recommend prioritizing a systematic clinical reasoning model with explicit reflective steps when using serious games for teaching clinical reasoning.

## Introduction

Clinical reasoning (CR) is increasingly important for healthcare professionals due to multiple factors affecting their daily work, including multimorbidity [1], technological advances [2] and time constraints [3]. At the same time, teaching (future) healthcare professionals CR skills is complex and time consuming [4,5]. Game-based learning (GBL) methods may offer opportunities to enrich teaching CR skills in a safe environment [6]. An overview of currently available published games designed to support the teaching of various aspects of CR may offer health professions educators new insights into CR education.

CR is a core competence in health professions that entails a patient-specific problem-solving process [7]. To support learners in mastering this complex skill, the CR process can be structured into systematic steps that incorporate logical considerations [8]. Adopting this cycle of steps aims not only to facilitate but also reveals the cognitive analytical process underlying CR. Each step systematically guides learners, leading to a decision that prioritizes the patient’s best interests in a specific situation. These CR steps include: 1) consider the patient situation, 2) collect cues, 3) process the information, 4) understand the patient problem, 5) set goals, 6) implement interventions, 7) evaluate outcomes, and 8) reflect and learn from these processes [8]. The explicit integration of reflection as a final step aligns with the overarching conceptual framework of reflective practice [9]. Emphasized as crucial for learning CR skills, reflection enables learners to analyze new experiences, identify patterns, and justify their CR practices [10]. This process effectively builds knowledge and experience, empowering novice learners to minimize inaccurate thinking [11] and enhance their CR skills [12]. Schön (1983) further emphasizes the importance of reflection as a crucial component for professionals to actively learn in practice, introducing the term ‘reflective practice’ [9].

The traditional approach for developing CR skills is through clinical environment learning. Although learners could benefit from real-life situations [13], this approach has several drawbacks, including a limited number and variety of patient cases [14] and variable supervision quality [15]. Furthermore, given the potential serious consequences of misdiagnosis [16], it may result in harmful situations for both students and patients. To address this issue, simulation and GBL provide modern and engaging teaching methods that replicate real-life scenarios in the classroom [17]. These methods can potentially expand knowledge-based teaching by promoting active, immersive, experimental, and safe learning experiences for both learners and patients [17,18].

Additionally, the incorporation of serious games in GBL surpasses the scope of mere simulation, resulting in a dynamic and enjoyable learning environment. Serious games, hereafter referred to as games, are defined as structured forms of play intended for objectives beyond entertainment [19]. They benefit learning by enhancing learner motivation and engagement toward achieving educational outcomes [20,21]. Game elements like clear goals, instant feedback, and adaptable challenges, can potentially foster a state of flow in learners, by which is meant a state of complete immersion and focus [20]. The integration of games in health professions education has gained significant attention [22,23]. Various game genres, including simulation games [6], board games [24], and escape rooms [25], are employed to foster the development of CR skills among learners. However, to date (2022), a comprehensive overview of available games, their characteristics, and their capacity to facilitate various steps of the CR cycle within the context of reflective practice remains absent [5].

With this scoping review we aim to provide an overview of currently published games designed to support the teaching of CR skills to (future) healthcare professionals. The study seeks to identify potential gaps through the lens of the CR cycle, with a specific interest in reflective practice. It concentrates on game characteristics and explicit incorporation of CR steps. The provided insights may aid individual health professions educators in understanding how games can be used to support CR education.

## Methods

### Design

A scoping review was conducted to assess the quantity, diversity, and nature of research on games for CR. Choosing a scoping review over a systematic review aligns with our constructivist perspective, emphasizing the exploration of a broad understanding rather than a comprehensive assessment to identify the best game for teaching CR [26,27]. Following Khalil and colleagues’ evidence-based approach for scoping reviews [28], we performed the following steps: 1) identifying the research question, 2) identifying relevant studies, 3) study selection, 4) presenting the data, and 5) collating the results. Results are reported in accordance with the Preferred Reporting Items for Systematic Review and Meta-analysis extension for Scoping Reviews (PRISMA-ScR) [29]. In addition, the authors of included articles were contacted to obtain any necessary additional data for the general overview.

### Search strategy

The literature search encompassed three stages to balance feasibility, extent, and comprehensiveness. In the first stage, we conducted a limited search in PubMed and CINAHL. The review team comprised three authors (GK, MH and TvH) and one additional colleague (LV). Two reviewers (GK, LV) independently screened titles and abstracts using the keywords ‘Clinical Reasoning’ AND ‘Game-Based Learning’. Hereafter, a review team meeting (GK, MH, TvH and LV) was held to discuss the first stage and introduce new keywords. In the second stage, conducted on June 17 (2022), we used the identified keywords as search terms across multiple databases, including PubMed, CINAHL, ERIC, PsycINFO, Scopus, Web of Science, and Embase. Coverage of health professions education literature was ensured thanks to this database selection [30]. The publication year was limited to January 2012 to June 2022, aligning with the developmental phase of game-related publications in health education [31]. Detailed search syntax information is available in the Supporting Information (Appendix A). For the third stage, in addition to Khalil et al.’s (2016) approach, we employed forward and backward snowballing to identify additional studies [32].

### Study selection

**Figure 1.**
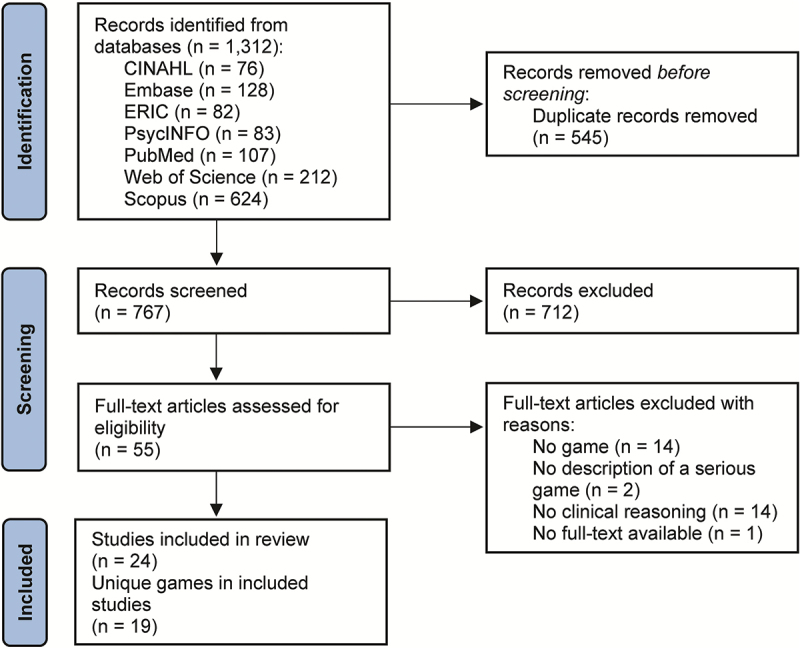
Flow diagram for the scoping review process adapting to the PRISMA-ScR.

Rayyan (Qatar Computing Research Institute, Doha, Qatar) was used to deduplicate and select articles [33]. After importing 1,312 articles into Rayyan, duplicates were automatically identified and eliminated. Manual deduplication by one reviewer (GK) followed to eliminate the remaining potential duplicates and ensure accuracy. The review process, as outlined in Figure 1, started with two reviewers (GK, LV) independently screening titles and abstracts using predefined criteria as described below. To maintain consistency, the process and criteria were discussed with the review team (GK, MH, TvH, and LV) after reviewing 10 articles. Studies were included if they (i) described games in the main manuscript, supplemental data, and/or online supplemental data, (ii) aimed to teach CR, and (iii) were written in English. Selected games required defined rules, challenges, and a goal-oriented or identifiable end state to differentiate them from mere simulations. In the context of CR, games had to be patient-specific and incorporate at least one CR cycle step. The choice of the CR cycle was based on its association with reflection as a crucial aspect of learning CR in simulation-based education [34]. If inclusion or exclusion was unclear based on the title and/or abstract, full text was retrieved. If uncertainties remained at this point, a third author (MH or TvH) served as referee for consensus. In the final step, both reviewers (GK, LV) independently reviewed all included papers and described games, with referee (MH) resolving interpretation discrepancies.

### Data synthesis

Game characteristics, technical requirements, and included steps of the CR cycle were extracted by analyzing all included papers. To increase reliability and consistency, two reviewers (GK, MH) evaluated the CR steps using a standardized table (Appendix C). The standardized table underwent testing and refinement by three authors (GK, MH, TvH) using five articles to ensure accurate data capture.

### Additional data

To address incomplete or insufficient descriptions of game characteristics in the articles, the authors from each study were sent an email with a request to provide additional data. The requested information included the game name, game population, case topics, number of cases, technical requirements, costs, and access. We distinguish the authors’ additions in the table of Appendix B by using *italicized* text.

## Results

A total of 1,312 articles were identified from seven databases, snowballing yielded no additional studies. After deduplication, 767 articles remained, of which 24 were included in this study (Figure 1), describing 19 unique games for teaching CR. Table 1 exhibits the key game characteristics, with a more detailed description available in Appendix B.

**Table 1.** Key characteristics of games for teaching clinical reasoning.

First author, publication year (Country)	Genre	Health profession	CR steps*	Case topic example	Language(s)
Besse, 2020 (Canada) [35]	Simulation	Nursing	1,2,3,4,5,6,7,8	COPD	English
Clauson, 2019 (US) [36]	Escape room	Pharmacy	1,2,3,4,5,6,7,8	Pharmacy	English
Gabriel, 2021 (US) [37]	Escape room	Nursing	1,2,3,4,6,7,8	Sepsis	English
Kubin, 2020 (US) [25]	Escape room	Nursing	1,2,4,5,6,7,8	Endocrine disorders	English
Koivisto, 2016 (Finland) [22]	Simulation	Nursing	1,2,3,4,5,6,7	Emergency setting	English and Finnish
Havola, 2021 (Finland) [38]					
Fonteneau, 2020 (France) [39]	Simulation	Medicine	1,2,3,4,6,7	Exacerbation of asthma	French
Korenoski, 2021 (US) [40]	Escape room	Pharmacy	1,2,4,5,6,7	Acetaminophen toxicity	English
Chon, 2019 (Germany) [41]	Simulation	Medicine	1,2,3,4,6,7	Cardiology	German
Middeke, 2018 (Germany) [23]					
Middeke, 2020 (Germany) [42]					
Raupach, 2021 (Germany) [43]					
Pisano, 2020 (US) [24]	Puzzle	Medicine	1,2,3,4,6,7	Anaemia	English
Zehler, 2021 (US) [44]	Puzzle	Nursing	1,3,4,6,7,8	Postpartum Hemorrhage	English
Blanié, 2020 (France) [45]	Simulation	Nursing	1,2,3,6,7	Brain trauma	French
Calik, 2022 (Turkey) [46]	Simulation	Nursing	1,2,5,6,7	Diabetic ketoacidosis	Turkish
Johnsen, 2016 (Norway) [47]	Simulation	Nursing	1,2,3,6,7	COPD	Norwegian
Johnsen, 2018 (Norway) [48]					
Sullivan, 2016 (US) [49]	Simulation	Medicine	1,2,3,6,7	Acute diverticulitis	English
Luu, 2020 (US) [50]	Simulation	Medicine	1,2,6,7	Pediatric Emergency Setting	English
Tyo, 2021 (US) [51]	Board game	Nursing	1,3,4,8	Respiratory failure	English
Smith, 2021 (US) [52]	Escape room	Nursing	1,6,7	Wound infection	English
Smith, 2021 (US) [53]	Escape room	Nursing	1,6,7	Pharmacology	English
Wu, 2021 (Taiwan) [54]	Simulation	Dentistry	7	Tooth composite resin filling	Mandarin Chinese

*Note*: Games are ordered by the number of included clinical reasoning steps. Detailed game characteristics can be found in Appendix B.

^*^The eight steps for clinical reasoning as described by Levett-Jones et al. [8] include: 1) consider the patient situation, 2) collect cues, 3) process information, 4) understand the patient problem, 5) set goals, 6) implement interventions, 7) evaluate outcomes, and 8) reflect and learn. The rationale for scoring CR steps per game is explained in Appendix C. When multiple authors address the same game, game characteristics are consolidated in the table, and the maximum number of clinical reasoning steps are shown.

### Descriptive findings

Most of the games were developed in North America (*n* = 12), specifically in the United States (*n* = 11) and Canada (*n* = 1) (Table 1). Most European games were developed in Northern European countries (*n* = 5). Only one of the games was developed in Asia, namely Taiwan. Games were predominantly in English (*n* = 13), while two were in French (*n* = 2) and one in Mandarin Chinese. Regarding health professions, the games included were mostly designed for Nursing (*n* = 11) as compared to Medicine (*n* = 5), Pharmacy (*n* = 2), and Dentistry (*n* = 1). Simulation games (*n* = 10) and escape rooms (*n* = 6) were the most common game genres, followed by puzzle (*n* = 2) and board games (*n* = 1).

### Insights by game genre

The majority of simulation games required individual play (*n* = 9) on digital devices with internet access (*n* = 9) and originated from Europe (*n* = 6) (Appendix B). Only one simulation game offered a virtual reality option. The number of cases per game ranged from one to forty-six, with only two simulation games allowing players to manage multiple patients simultaneously. All escape rooms, puzzle games, and board games required team play (*n* = 9), primarily without the use of digital devices (*n* = 8), and were developed in North America (*n* = 9). The number of cases provided in escape rooms, puzzle games, and board games ranged from one to thirteen, with only one escape room allowing players to manage multiple patients simultaneously.

### Clinical reasoning

Among the unique games (*n* = 19), only two incorporated all eight steps in Levett-Jones’ eight step CR circle [8]. Most games included considering the patient situation (step 1; *n* = 18), collecting cues (step 2; *n* = 15), implementing interventions (step 6; *n* = 17), and evaluating outcomes (step 7; *n* = 18). Setting goals (step 5; *n* = 7) and reflecting and learning from processes (step 8; *n* = 6) were least integrated. Notably, only four games described using the CR cycle as an educational model [8].

### Additional info from authors

A total of 15 authors of the 24 included studies responded. Among them, three authors mentioned receiving additional educator training prior to gameplay. Regarding game access availability, three games were openly accessible, three required access requests, six were not openly accessible, and seven games did not explicitly report accessibility status.

## Discussion

In this review, we identified 19 games that demonstrated variations in game characteristics and incorporation of CR steps. Simulation games and escape rooms emerged as predominant game genres, differing in technological requirements, number of patient cases, opportunities for managing multiple patients, and individual or team play design. Most games did not incorporate all eight steps of the CR cycle [8], hindering the explicit presentation of the CR process and impact on reflective practice. Moreover, when reflection (step 8) did take place, it predominantly took the form of implicit self-reflection based on provided feedback and outcomes within the game. Guided reflection during post-gameplay standalone debriefing was infrequently used, thereby constraining the full potential of the learning experience.

### Exploring simulation games and escape rooms for clinical reasoning

In a prior scoping review [31] investigating the learning efficiency of games in health professions education, various genres were explored, including action games for technical skill practice and simulation games for cognitive skill improvement. In contrast, our review focused on mapping games for teaching CR, with 16 of the 19 games being simulation games and escape rooms. Future game genre selection should consider stakeholder preferences and contextual factors.

Simulation games merge gameplay with educational objectives, allowing players to embody the role of healthcare practitioners in virtual clinical scenarios [6]. Learners can develop CR skills through repeated practice in various patient situations [23]. The simulation games in our study primarily supported individual online play, ensuring convenient accessibility with electronic devices and reliable internet connectivity. However, challenges such as increased screen time, effective technology use, and technology fairness need to be addressed [55].

Escape rooms are designed to foster learning while developing teamwork and collaboration skills [56]. Consistent with our findings, all identified escape rooms required players to participate in teams. Despite often being one-offs, learners practice problem-solving and teamwork while unravelling puzzles and progressing within a specified time limit [56]. Implementing educational escape rooms poses challenges, including preparation, educator facilitation, and learner stress due to time constraints [57].

Educators and program developers should consider aligning game genres with educational goals and context. For example, escape rooms often offer single-use experiences, while most simulation games can be replayed over and over. Moreover, variations in individual or team play should be considered, with individual play benefiting learning in simple tasks and teamwork in more complex problem-solving tasks [58].

### Clinical reasoning and reflection

According to the recommendation of the European Consortium to enhance explicit clinical reasoning (CR) teaching approaches [59], our review identified that only two games explicitly described the CR cycle [8] as an analytical learning model. Moreover, a minority of games in our review (2 out of 19) incorporate all steps of the CR cycle, with goal setting (step 5) and reflection and learning (step 8) being the least prevalent (Appendix C).

Conceptualizing CR is challenging [59] as it encompasses analytic and conscious reasoning (system 2) and non-analytical and intuitive reasoning (system 1), according to cognitive psychology [60]. Experts, unlike novices, operate at an advanced stage in the development of CR skills, primarily due to their extensive knowledge and exposure to prior experiences. Consequently, experts rely on intuition when facing familiar problems, occasional supplemented by analytic thinking when encountering unfamiliar and complex problems [60]. The choice between these forms may be context-depended, given the diverse prior knowledge and experiences of (future) healthcare professionals [4].

In the realm of CR education, various approaches can be considered, taking into account both analytical and non-analytical, or a combination of both, forms of reasoning [4]. Games predominantly operate in the analytical field of reasoning, likely due to their pre-programmed nature. While non-analytic reasoning can coexist with analytic reasoning [4,7], games may face challenges in encompassing the non-analytic approach for every player. Therefore, games should be seen as supportive rather than a standalone method for teaching CR. However, the dichotomy’s black-and-white nature of system 1 and 2 thinking is questioned, with critics like Melnikoff and Bargh (2018) warning against oversimplifying specific dual-process models [61]. In our scoping review, we deliberately avoided this dichotomy and searching for best practices in CR teaching. Instead, we explore games, mainly in the analytical field, through the CR cycle lens to support teaching CR.

While reflection is not explicitly facilitated in most games, Koivisto et al.’s (2016) simulation game demonstrated that its feedback can induce reflection through self-debriefing. Self-debriefing is a flexible approach that allows learners to reflect on their performance, identify strengths and weaknesses, and develop an improvement plan on their own [62]. Learners can engage in self-debriefing independently or use pre-set questions to further refine their self-reflection process. This approach holds the potential to elevate self-directed learning skills, including self-awareness, goal setting, and critical thinking skills [63]. However, it is noteworthy that novice learners may benefit from additional guidance and support as their self-directed learning skills are still developing [64].

One promising approach is combined debriefing, which integrates self-debriefing with instructor-led debriefing. This approach offers learners the opportunity to clarify their thoughts on the CR process and formulate relevant questions before receiving structured instructor feedback [65]. Research underscores that combined debriefing not only amplifies learners’ self-awareness, but also deepen their understanding of the learning experience [66] and enhances their problem-solving skills [67]. Nevertheless, determining whether self-debriefing alone is sufficient or if additional instructor-led debriefing is necessary for specific learners remains to be determined.

Interestingly, in simulation-based education, instructor-led debriefing is a widely recognized method to enhance CR skills [68,69]. However, our findings indicate that only 31.6% of the games include instructor-led debriefing. Although recognized best practices for simulation debriefings exist [70], literature on the proper application of different debriefing methods in the context of serious gaming lacks consistency [71]. Therefore, further research is required to identify effective game debriefing techniques and establish best practices in this setting, ultimately enhancing the capacity to facilitate reflective practice.

### Strengths and limitations

Our study provides insights into the landscape of serious games for CR, by evaluating the integration of the CR cycle and reflective practice in games, and addressing gaps in the literature. The review process was conducted independently by two researchers, aiming to enhance the reliability of the findings. Missing game characteristics were gathered by contacting the authors of the included articles, enhancing the comprehensiveness of the analysis.

Simultaneously, some limitations apply: firstly, this study focused on games published in English language peer-reviewed journals and conference proceedings, potentially overlooking unpublished games. Also, the data analysis relies on descriptions in scientific articles, introducing the possibility of confirmation bias due to a lack of direct engagement and hands-on experiences with the games in health professions education. Secondly, only a small number of included articles used the CR cycle as described by Levett-Jones et al. (2010) as an educational model for CR. Not explicitly mentioning the CR steps in an article, however, does not imply their absence in the game. Furthermore, various models for teaching CR exist, and alternative models may be equally effective in mapping CR steps in the games.

### Implications for education and future research

This study offers educators and program developers insights into the potential of games for teaching CR in health professions education, although it does not directly address educational benefits. Teaching CR involves considerations beyond cognition, including context, emotions, and institutional factors when selecting suitable games [7]. Moreover, despite existing escape room design guidelines [72], there is still a lack of guidance for both game developers and educators to fully harness the learning potential of games in health professions education. This emphasizes the need for further research to identify appropriate educational strategies and validate the claimed educational benefits. An avenue for exploration involves applying debriefing techniques from simulation education [63] in GBL and assessing the effectiveness of reflection in GBL.

Game-developers should prioritize the incorporation of debriefing and reflection as integral components of the learning process. The escape room outlined by Gabriel et al. (2021) stands out as a notable example, as it encourages learners to explain their decision-making rationale to instructors, effectively integrating reflection-in-action within the gaming experience. By offering debriefing options, game developers can potentially enrich the learning experience.

## Conclusion

Our study identified 19 games that can be used by educators to teach CR, with simulation games and escape rooms as prominent game genres. When selecting games to teach specific CR steps, careful consideration of contextual and institutional factors is essential. While this review provides an overview of the current games for CR and their characteristics, further research is needed to fully explore their effective use in health professions education and unlock their full potential for enhancing the learning experience. Furthermore, most games do not explicitly incorporate all the steps of the CR cycle, suggesting a potential limitation in terms of fully developing CR without incorporating intentional reasoning errors. Notably, reflection, a crucial step in learning CR and integral to reflective practice, is a poor relation in most included games. Since self-debriefing is a skill that has to be developed over time, combined debriefing can contribute to the learning process of developing CR. Therefore, we recommend prioritizing the utilization of a systematic CR model with reflective steps when promoting games for teaching CR.

## Supplementary Material

Supplemental Material
