# Supplementary material for: Games to support teaching clinical reasoning in health professions education: a scoping review
Source: Med Educ Online. 2024 Feb 23;29(1):2316971. doi: 10.1080/10872981.2024.2316971 (PMC10896137; doi:10.1080/10872981.2024.2316971)
Supplement: Supplemental Material [file ZMEO_A_2316971_SM1591.docx]

*Appendix A: Search strings for each data base*

Search string PubMed:

((Gamification [Mesh] OR gamification [tiab] OR “game-based learn*” [tiab] OR “serious gam*” [tiab] OR “simulation gam*” [tiab] OR “virtual realit*” [tiab] OR “augmented realit*” [tiab] OR “mixed realit*” [tiab] OR “escape room*” [tiab] OR “video gam*” [tiab] OR “electronic gam*” [tiab] OR “computer gam*” [tiab] OR “virtual gam*” [tiab] OR “board gam*” [tiab] OR “game-based application*” [tiab]) AND (Clinical Reasoning [Mesh] OR “clinical reasoning” [tiab] OR “clinical judgment*” [tiab] OR “critical think*” [tiab] OR “critical reflection” [tiab])) AND (2012:2022[pdat])

Search string Web of Science:

(TI=(“gamification” OR “game-based learn*” OR “serious gam*” OR “simulation gam*” OR “virtual reality” OR “augmented reality” OR “mixed reality” OR “escape room*” OR “video gam*” OR “electronic gam*” OR “computer gam*” OR “virtual gam*” OR “board gam*” OR “game-based application*”) OR AB=( “gamification” OR “game-based learn*” OR “serious gam*” OR “simulation gam*” OR “virtual reality” OR “augmented reality” OR “mixed reality” OR “escape room*” OR “video gam*” OR “electronic gam*” OR “computer gam*” OR “virtual gam*” OR “board gam*” OR “game-based application*”)) AND (TI=(“Clinical Reasoning” OR “clinical judgment*” OR “critical thinking” OR “critical reflection”) OR AB=(“Clinical Reasoning” OR “clinical judgment*” OR “critical thinking” OR “critical reflection”)) AND (PY=(2012-2022))

Search string (EBSCOhost) CINAHL & ERIC & PsycINFO:

((MH “Gamification” OR TI gamification OR TI “game-based learn*” OR TI “serious gam*” OR TI “simulation gam*” OR TI “virtual reality” OR TI “mixed reality” OR TI “augmented reality” OR TI “escape room*” OR TI “video gam*” OR TI “electronic gam*” OR TI “computer gam*” OR TI “virtual gam*” OR TI “board gam*” OR TI “game-based application*” OR AB gamification OR AB “game-based learning” OR AB “serious gam*” OR AB “simulation gam*” OR AB “virtual reality” OR AB “mixed reality” OR AB “augmented reality” OR AB “escape room*” OR AB “video gam*” OR AB “electronic gam*” OR AB “computer gam*” OR AB “virtual gam*” OR AB “board gam*” OR AB “game-based application*”) AND (MH “Clinical Reasoning” OR TI “clinical reasoning” OR TI “clinical judgment*” OR TI “critical thinking” OR TI “critical reflection” OR AB “clinical reasoning” OR AB “clinical judgment*” OR AB “critical thinking” OR AB “critical reflection”)) AND (PY 2012-2022)

Search string Scopus:

TITLE-ABS-KEY(( {Gamification} OR {game-based learning} OR {game-based learned} OR {serious game} OR {serious games} OR {serious gaming} OR {simulation game} OR {simulation games} OR {simulation gaming} OR {virtual reality} OR {virtual realities} OR {augmented reality} OR {augmented realities} OR {mixed reality} OR {mixed realities} OR {escape room} OR {escape rooms} OR {video game} OR {video games} OR {video gaming} OR {electronic game} OR {electronic games} OR {electronic gaming} OR {computer game} OR {computer games} OR {computer gaming} OR {virtual game} OR {virtual games} OR {virtual gaming} OR {board games} OR {board game} OR {board gaming} OR {game-based application} OR {game-based applications}) AND ({Clinical Reasoning} OR {clinical judgment} OR {clinical judgments} OR {critical thinking} OR {critical reflection} OR {critical reflections})) AND PUBYEAR > 2012

Search string EMBASE:

(‘gamification’/exp OR ‘gamification’ OR ‘gamification’:ab,ti OR ‘game-based learn*’:ab,ti OR ‘serious gam*’:ab,ti OR ‘simulation gam*’:ab,ti OR ‘virtual realit*’:ab,ti OR ‘augmented realit*’:ab,ti OR ‘mixed realit*’:ab,ti OR ‘escape room*’:ab,ti OR ‘video gam*’:ab,ti OR ‘electronic gam*’:ab,ti OR ‘computer gam*’:ab,ti OR ‘virtual gam*’:ab,ti OR ‘board gam*’:ab,ti OR ‘game-based application*’:ab,ti) AND (‘clinical reasoning’/exp OR ‘clinical reasoning’ OR ‘clinical reasoning’:ab,ti OR ‘clinical judgment*’:ab,ti OR ‘critical think*’:ab,ti OR ‘critical reflection’:ab,ti) AND [2012-2022]/py

*Appendix B:* *Characteristics of games for teaching clinical reasoning*

| **Game name(s)** | **Author(s), Publication year (Country)** | **Game description** | **Health Profession, Game population** | **Step(s) in CR cycle*** | **Case topic(s) (Number of cases)** | **Game's language(s)** | **Type of study** | **Technical requirements** | **Game costs and access** | **Additional training for educators** |
| --- | --- | --- | --- | --- | --- | --- | --- | --- | --- | --- |
| *A Day in the Endocrine and Musculoskeletal Urgent Care Center* | Kubin, 2020 (US)^21^ | A serious escape room, played in a classroom, in which students work in teams to escape three different boxes by solving puzzles and riddles related to assessing and diagnosing the patient and selecting appropriate interventions for four patients at the same time, while getting in-game feedback and debriefed afterwards. | Nursing, *4th year undergraduates* | 1,2,4,5,6,7,8 | - Endocrine disorders in a pediatric acute care center *(3)* - Musculoskeletal disorders in a pediatric acute care center *(2)* | - English | Quasi-experimental | *- Computer and printer* | Full game: - Costs: *limited to the supplies for the escape activity, The total cost for supplies was $275.00* - Open access: *n/a* Demo: *n/a* | *No additional training or support is required; escape room creation resources are provided* |
| Bringing it to the Bedside (BITTB) | Besse et al., 2020 (Canada)^29^ | A serious simulation game, played in a simulation capstone lab (not online), in which students must work in teams to manage patient situations for a single patient at the time, while getting in-game feedback and debriefed afterwards. | Nursing, 1st year undergraduates | 1,2,3,4,5,6,7,8 | - COPD/dyspnea (1) - Hypertension (1) - Postoperative with diabetes (1) *- Head/arm injury (1)* | - English | Pre-experimental design | *None* | Full game: - Costs: *limited to the supplies for the simulation* - Open access: *on request* Demo: *n/a* | *Lab instructors received an orientation to the BITTB game (1 hour) and then each of the authors acted as facilitator / mentors for the first round of the game. Trained instructors serve as mentors in future offerings of BITTB* |
| - *CareMe®  Metropolia University of Applied Sciences*  - *Virtual Reality Simulation* | Koivisto et al., 2016 (Finland)^18^ | A serious online computer-based simulation game, played with or without VR headset, in which students individually must select appropriate history questions, patient examinations, and treatments for a single virtual patient at the time, while getting in-game feedback. | Nursing, 2nd year undergraduates | 1,2,3,4,5,6,7 | Simulation game:  - Home healthcare settings (not explicitly reported)  - Surgical Nursing (not explicitly reported)  - Surgical (not explicitly reported) - Internal medical (not explicitly reported) - Emergency (not explicitly reported)  VR: - Chest pain and resuscitation (1) | - Finnish - English | Cross-sectional descriptive | - Computer - Internet | Full game: - Costs: not explicitly reported - Open access: *no* Demo: - Costs: not explicitly reported - Open access: *no* | *None* |
|  | Havola et al., 2021 (Finland)^32^ |  | Nursing, *3rd and 4th year undergraduates* | 1,2,3,6,7 |  |  |  | - Virtual Reality headset |  |  |
| Effic’Asthme | Fonteneau et al., 2020 (France)^33^ | A serious online simulation game, played on a mobile phone, in which students/parents must individually observe a child's avatar to detect signs and select appropriate actions to manage a single virtual patient at the time, while getting in-game feedback. | Medicine, 5th year undergraduates | 1,2,3,4,6,7 | - Exacerbation of asthma in a child aged 12-months *(6)* | - French | Quasi-experimental | *- Mobile phone or tablet* | Full game: - Costs: *free* - Open access: *yes,* [*https://play.google.com/store/apps/details?id=fr.parisdescartes.efficasthme&hl=en_US*](https://play.google.com/store/apps/details?id=fr.parisdescartes.efficasthme&hl=en_US)  Demo: *n/a* | *None* |
| EMERGE | Middeke et al., 2018 (Germany)^19^ | A serious online computer-based simulation game in which students must individually select appropriate actions to complete history taking and patient management for up to ten virtual patients with different diseases at the same time, while getting in-game feedback. | Medicine, 3rd, 4th, 5th, and 6th year undergraduates | 1,2,4,6,7 | - Cardiology /pulmonology (12)  - Nephrology /rheumatology (11)  - Gastroenterology /endocrinology (12)  - Haematology /oncology (11) | - German | Quasi-experimental | - Computer - Internet | Full game: - Costs: not explicitly reported - Open access: *no* Demo: - Costs: not explicitly reported - Open access: <http://elearning.uke.de/HOOU/EFEWebGLCrunching/> | *Not done in this trials but needed if implemented elsewhere* |
|  | Chon et al., 2019 (Germany)^35^ |  |  | 1,2,4,6,7 |  |  |  |  |  |  |
|  | Middeke et al., 2020 (Germany)^36^ |  |  | 1,2,3,4,6,7 |  |  |  |  |  |  |
|  | Raupach et al., 2021 (Germany)^37^ |  |  | 1,2,4,6,7 |  |  |  |  |  |  |
| Escape the Sepsis Room | Gabriel et al., 2021 (US)^31^ | A serious escape room in which nurses work in teams to escape the room by solving puzzles and riddles related to assessing and diagnosing a single case patient at the time, while getting in-game feedback and debriefing and debriefed afterwards. | Nursing, *graduates and experts* | 1,2,3,4,6,7,8 | - Sepsis (1) | - English | Quasi-experimental | Not explicitly reported | Full game: - Costs: not explicitly reported - Open access: not explicitly reported Demo: - Costs: not explicitly reported - Open access: not explicitly reported | Not explicitly reported |
| “Jeg får ikke puste” (I cannot breathe) | - Johnsen et al., 2016 (Norway)^41^ | A serious online computer-based simulation game in which students individually must select the correct actions of a video-recorded nurse when taking history and performing examinations for a single virtual patient at the time, while getting in-game feedback. | Nursing, 2nd year undergraduates | 1,2,6,7 | - COPD in a home healthcare (1)  - COPD in home healthcare setting (2)  - COPD in hospital setting (2) | - Norwegian | Descriptive | - Computer - Internet | Full game: - Costs: not explicitly reported - Open access: *on request* Demo: Not explicitly reported | Not explicitly reported |
|  | Johnsen et al., 2018 (Norway)^42^ |  |  | 1,2,3,6,7 |  |  |  |  |  |  |
| LabForGames Warning | Blanié et al., 2020 (France)^39^ | A serious online computer-based simulation game in which students must individually select appropriate actions regarding history taking, clinical exams, care report writing and calling the physician for a single virtual patient at the time, while getting in-game feedback. | Nursing, 2nd year undergraduates, recent graduates and experts | 1,2,3,6,7 | - Post-operative hemorrhage (1) - Brain trauma (1) - Obstructed intestinal tract (1) | - French | Quasi-experimental | - Computer - Internet | Full game: - Costs: not explicitly reported - Open access: not explicitly reported Demo: - Costs: not explicitly reported - Open access: not explicitly reported | Not explicitly reported |
| Medical-Surgical Clue | Tyo and McCurry, 2021 (US)^45^ | A serious board game, played in a classroom, in which students work in teams to interpret assessment data, vital signs, and diagnostic results to determine which suspect illness murdered the victim, while debriefed afterwards. | Nursing, 1st year postgraduates | 1,3,4,8 | - Acute respiratory failure (1) - Acute alcohol withdrawal (1) - Sepsis (1) - Septic shock (1) - Chest tube malfunction (1)  - Hypovolemic shock (1) - Pulmonary embolism or acute respiratory distress syndrome (1) | - English | Descriptive | Not explicitly reported | Full game: - Costs: not explicitly reported - Open access: not explicitly reported Demo: - Costs: not explicitly reported - Open access: not explicitly reported | Not explicitly reported |
| Minute to Win It | Zehler and Musallam, 2021 (US)^38^ | A serious puzzle game, played in a classroom, in which students work in teams to complete game activities related to cue recognition, hypothesis formulation and prioritization, and evaluating interventions and patient responses, while getting in-game feedback and debriefed afterwards. | Nursing, *3rd year undergraduates* | 1,3,4,6,7,8 | - Postpartum Hemorrhage *(1)* | - English | Quasi-experimental | *- Computer or laptop with screen* | Full game: - Costs: *$75* - Open access: *no* Demo: *n/a* | *None* |
| Not explicitly reported | Korenoski et al., 2021 (US)^34^ | A serious escape room, played in a classroom, in which students work in teams to escape by solving puzzles and riddles related to assessing and diagnosing the patient and selecting appropriate interventions for a single patient at the time, while getting in-game feedback. | Pharmacy, 3rd year undergraduates | 1,2,4,5,6,7 | - Lithium toxicity (1) - Acetaminophen toxicity and serotonin syndrome (1) | - English | Quasi-experimental | Not explicitly reported | Full game: - Costs: The university had already purchased the equipment, so no additional costs were associated with the activity - Open access: not explicitly reported Demo: - Costs: not explicitly reported - Open access: not explicitly reported | Not explicitly reported |
| *Nursing Game* | Calik and Kapucu, 2022 (Turkey)^40^ | A serious online computer-based simulation game in which students must individually select appropriate options regarding patient history and patient examinations for a single virtual patient at the time, while getting in-game feedback. | Nursing, 2nd year undergraduates | 1,2,5,6,7 | - Diabetic ketoacidosis (1) | - Turkish | Randomized Controlled Trial | - Computer, *laptop or mobile phone* - Internet | Full game: - Costs: *free* - Open access: *yes,* [*https://calikafra.itch.io/nursing-game*](https://calikafra.itch.io/nursing-game)  Demo: *n/a* | *None* |
| *Pharmacology Review Escape Room* | Smith and Davis, 2021 (US)^47^ | A serious online escape room in which nurses work in teams to escape the room by solving puzzles and riddles related to assessing and diagnosing a single patient at the same time while getting in-game feedback. | Nursing, 4th year undergraduates | 1,6,7 | - Pharmacology *(5)* | - English | Descriptive cross-sectional | *- Computer or laptop - Internet* | Full game: - Costs: none - Open access: *limited to students and educators who have the link.* Demo:  *On request* | *None* |
| *Pharmacy Escape Game* | Clauson et al., 2019 (US)^30^ | A serious escape room in which students work in teams to escape the room by gathering and assessing patient information and creating a patient care plan for a single patient at the time, while getting in-game feedback and debriefed afterwards. | Pharmacy, *graduates* | 1,2,3,4,5,6,7,8 | - Ambulatory care pharmacy (1) - Community pharmacy (1) - Inpatient pharmacy (1) | - English | Quasi-experimental | *None* | Full game: - Costs: *$400* - Open access: not explicitly reported Demo: *n/a* | *Cases, Room design, and prompts were reviewed with each instructor* |
| The Bloody Board Game | Pisano et al., 2020 (US)^20^ | A serious puzzle game, played in a classroom, in which students work in teams to earn money by correctly answering questions in order to pay for diagnostic tests required for patient workup while getting in-game feedback. | Medicine, 1st, 2nd, and 3rd year postgraduates | 1,2,3,4,6,7 | - *Anaemia (13)* | - English | Quasi-experimental | *- Computer with screen (or printer/dry erase board) - Internet* | Full game: - Costs: *free.* - Open access: *yes,* [*https://www.mededportal.org/doi/10.15766/mep_2374-8265.11057*](https://www.mededportal.org/doi/10.15766/mep_2374-8265.11057)  Demo: *n/a* | *None* |
| - The Bone Dry Escape Room - The Open Wide Escape Room - The All Choked Up Escape Room | Smith and Paul, 2021 (US)^46^ | Three serious escape room in which nurses work in teams to escape the room by solving puzzles and riddles related to assessing and diagnosing a single patient at the same time, while getting in-game feedback. | Nursing, 1st year undergraduates | 1,6,7 | - Diabetic ketoacidosis (1) - Wound infection (1) - Aspiration pneumonia (1) | - English | Descriptive cross-sectional | Not explicitly reported | Full game: - Costs: Not explicitly reported - Open access: Not explicitly reported Demo: - Costs: Not explicitly reported - Open access: Not explicitly reported | Not explicitly reported |
| Virtual Dental Clinic | Wu et al., 2021 (Taiwan)^48^ | A serious online computer-based simulation game in which students must individually apply their knowledge of dental treatment procedures to identify various instruments based on a given dental procedure for a single patient at the same time, while getting in-game feedback. | Dentistry, 5th year undergraduates | 7 | - Tooth composite resin filling (1) - Root canal therapy (1) - Cement mixing methods (2) | - Mandarin Chinese | -Descriptive - Mixed methods - Quasi-experimental | *- Computer or laptop* | Full game: - Costs: *free download for use* - Open access: *no* Demo: n/a | *None* |
| Virtual Surgical Patient Cases | Sullivan et al., 2016 (US)^43^ | A serious online computer-based simulation game in which students must individually select the right option in a decision tree to complete history taking, diagnosis, and treatment for a single patient at the same time, while getting in-game feedback. | Medicine, 3rd year undergraduates | 1,2,3,6,7 | - Acute diverticulitis (1) - Upper gastrointestinal bleeding (1) | - English | Quasi-experimental mixed method | *- Computer or laptop - Internet* | Full game: - Costs: *$300-$500 total per year* - Open access: *no* Demo:  - Costs: *no*  - Open access: *no* | *Instructors were able to access the case site to review the platform and students’ responses prior to the case discussion* |
| VitalSigns™ | Luu et al., 2020 (US)^44^ | A serious online computer-based simulation game in which students individually must triage, stabilize, discharge, or admit up to seven virtual patients with different diseases at the same time, while getting in-game feedback. | Medicine, pre-medical undergraduates, 2nd year undergraduates, graduates and experts | 1,2,6,7 | - Pediatric emergency department cases *(35)* | - English | Cross-sectional quasi-experimental | - Computer - Internet | Full game: - Costs: not explicitly reported - Open access: *no* Demo: - Costs: not explicitly reported - Open access: not explicitly reported | *None* |
| Note: Each game included in this review is presented with its corresponding game characteristics, technical requirements, and the steps of the clinical reasoning cycle that are incorporated.^8^ Additional data provided by the authors via email are indicated by *italicized* text.  *Rationale for scoring CR cycle steps in every game can be found in Appendix C. | | | | | |  |  |  |  |  |

*Appendix C: Rationale for scoring clinical reasoning cycle steps in every game*

| **Author(s), Publication year (Country)** | **step 1: consider facts** | **Justification (1)** | **step 2: collect info** | **Justification (2)** | **step 3: process info** | **Justification (3)** | **step 4: identify problem** | **Justification (4)** | **step 5: establish goals** | **Justification (5)** | **step 6: take action** | **Justification (6)** | **step 7: evaluate action** | **Justification (7)** | **step 8: reflect** | **Justification (8)** |
| --- | --- | --- | --- | --- | --- | --- | --- | --- | --- | --- | --- | --- | --- | --- | --- | --- |
| **Besse et al., 2020 (Canada)** | 1 | Players had access to 3 scenarios | 1 | Nursing assessment of vital signs, respiratory system, blood glucose, wound, pain, mental state | 1 | Critical thinking | 1 | “Developing a care plan based on observation of the scenario and reporting.” | 1 | “Developing a care plan based on observation of the scenario and reporting.” | 1 | Players received points for proficiency in interventions | 1 | Evaluators involved, discussing the actions throughout the simulation provided real-time feedback and direction to the players and ensured the scenario stayed on track | 1 | “Each scenario lasted 50 minutes, which included 25 minutes for the simulation and 25 minutes for the debriefing.” “If the Players missed a CTO [Critical Thinking Opportunity], the Game ShowHost could pause the game and ask them to think critically about their actions.” |
| **Blanié et al., 2020 (France)** | 1 | Players underwent a brief introduction of each of the 3 cases | 1 | “During the case, participants can perform different actions: history taking, clinical exams (circulatory assessment, neurologic assessment, skin temperature, etc.), care report writing and calling the physician.” | 1 | “During the case, participants can perform different actions: history taking, clinical exams (circulatory assessment, neurologic assessment, skin temperature, etc.), care report writing and calling the physician.” | 0 | Not explicitly reported | 0 | “During the case, participants can perform different actions: history taking, clinical exams (circulatory assessment, neurologic assessment, skin temperature, etc.), care report writing and calling the physician.” | 1 | Call physician | 1 | “At the end of each scenario, virtual automatic feedback was presented to the participant.” | 0 | No instructor-led reflection was provided |
| **Calik and Kapucu, 2022 (Turkey)** | 1 | Players were presented with one case (DKA) | 1 | Patient history, vital signs | 0 | Not explicitly reported | 0 | Not explicitly reported | 1 | Nursing care plan | 1 | Selecting assessment, vital signs, and laboratory tests | 1 | Evaluate action | 0 | No instructor-led reflection was provided |
| **Chon et al., 2019 (Germany)** | 1 | “When starting the simulation, students [the players] get information about the incoming patient from an emergency physician.” | 1 | “They [the players] dispatch the patient to an examination room, take the patient’s medical history, order diagnostic tests, and establish a diagnosis and treatment.” | 0 | Not explicitly reported | 1 | “They [the players] dispatch the patient to an examination room, take the patient’s medical history, order diagnostic tests, and establish a diagnosis and treatment.” | 0 | Not explicitly reported | 1 | “They [the players] dispatch the patient to an examination room, take the patient’s medical history, order diagnostic tests, and establish a diagnosis and treatment.” | 1 | “Patients respond to treatments and medications have effects and side effects.” “At the end of the game, students [the players] were debriefed by a virtual doctor, who informed them about the correct diagnosis and treatment for each patient.” | 0 | Informed about treatment, more feedback than reflection moment and therefore self-debrief |
| **Clauson et al., 2019 (US)** | 1 | Players were required to follow a patient throughout multiple steps | 1 | “Gather and assess multiple pieces of information in order to complete a health history on the patient.” | 1 | “Gather and assess multiple pieces of information in order to complete a health history on the patient.” | 1 | Identify renal dysfunction and medication error | 1 | “Create an appropriate patient care plan.” | 1 | Specific objectives in the escape room activity: “Initiate appropriate preventative inpatient treatment.” and “Appropriately dose medication for a patient with renal dysfunction.” | 1 | Through correcting puzzles and clues | 1 | “After each team completed the final room, the students [the players] received a debrief on the exercise.” |
| **Fonteneau et al., 2020 (France)** | 1 | “Each scenario starts with a short briefing of the situation.” | 1 | “Detect any sign of respiratory distress and to listen for a cough or wheezing.” | 1 | Decide on severity diagnosis and what medication to give | 1 | Establish severity asthma exacerbation | 0 | Not explicitly reported | 1 | “The user needs to choose the actions to be performed to manage the asthma exacerbation.” | 1 | “Once the scenario is completed, an automated, point-by-point debriefing is provided. Points are awarded for actions performed correctly, and an overall success rate of the mission out of 100% is given.” | 0 | No instructor-led reflection was provided |
| **Gabriel et al., 2021 (US)** | 1 | “A patient scenario was read to the groups of nurses [the players].” | 1 | “The nurses [the players] in the room asked questions” | 1 | “If the correct item was chosen, they had to explain to the moderators [the experts] the rationale for why they were picking that specific clue.” | 1 | “The final puzzle/clue entailed the nurses assessing the actual patient where they found the patient’s suspected source of infection. While performing a skin assessment, the nurses [the players] found a Stage IV pressure ulcer to the sacrum.” | 0 | Not explicitly reported | 1 | “The purpose of Escape the Sepsis Room was to increase nurses’ knowledge and confidence in early identification and management of sepsis in older adults.” | 1 | “If a distractor [wrong] item was chosen, the patient became increasingly more agitated and vital signs worsened.” | 1 | “Each team was debriefed using an outline provided by the Communication and Education Workgroup for consistency. This allowed those who did not solve all of the puzzles to leave with the correct information. Debriefing also provided time to ask questions and think critically about how to use CALF [mnemonic] to save Sam Septicemia [the [patient].” |
| **Havola et al., 2021 (Finland)** | 1 | Players receive a briefing on scenarios | 1 | “Assessment of the patient's clinical condition was made by interviewing and observing the patient and measuring vital signs.” | 1 | “Further, the objective was that students [the players] apply the ABCDE approach, which allows a systematic way to assess a patient's clinical condition.” | 0 | Not explicitly reported | 0 | Not explicitly reported | 1 | “In the scenarios, players assessed patient clinical condition and patient deterioration and made necessary nursing interventions.” | 1 | “The feedback given by the game system was immediate, sustained, and cumulative and was given as score, in the form of reasoning, patient reactions, answers via textbox, in-game facilitator comments, and success and failure effects. Moreover, at the end of each scenario, players could see their playing performance on a personalized playing analytics page.” | 0 | No instructor-led reflection was provided |
| **Johnsen et al., 2016 (Norway)** | 1 | Players undergo a brief introduction to the scenario, presented through a video-based introduction | 1 | Through quizzes and puzzles gathering information | 0 | Not explicitly reported | 0 | Not explicitly reported | 0 | Not explicitly reported | 1 | Performing actions | 1 | Direct embedded feedback in scenario | 0 | No instructor-led reflection was provided |
| **Johnsen et al., 2018 (Norway)** | 1 | Players have to select appropriate information to provide to the patient about his diagnosis and medication | 1 | Select appropriate information to provide to the patient about his diagnosis and medication | 1 | Questions formulated to enhance analyzation and transfer to scenario | 0 | Not explicitly reported | 0 | Not explicitly reported | 1 | Players need to select appropriate assessments and interventions | 1 | “When students [the players] submit their answer(s), they receive feedback from the nurse character in the scenario through a demonstration of the proper things to do or say.” | 0 | No instructor-led reflection was provided |
| **Koivisto et al., 2016 (Finland)** | 1 | Players were presented with several post-op scenarios | 1 | “The game guides learners through collecting and processing information, identifying problems/issues, establishing goals, taking action, and evaluating outcomes.” | 1 | “The game guides learners through collecting and processing information, identifying problems/issues, establishing goals, taking action, and evaluating outcomes.” | 1 | “The game guides learners through collecting and processing information, identifying problems/issues, establishing goals, taking action, and evaluating outcomes.” | 1 | “The game guides learners through collecting and processing information, identifying problems/issues, establishing goals, taking action, and evaluating outcomes.” | 1 | “The game guides learners through collecting and processing information, identifying problems/issues, establishing goals, taking action, and evaluating outcomes.” | 1 | “The systems provides for immediate, sustained, and cumulative feedback in the form of points, patient reactions, in game facilitator's comments, and success and failure effects.” | 0 | No instructor-led reflection was provided |
| **Korenoski et al., 2021 (US)** | 1 | “At the start of the case, students [the players] were given a brief medical history and reason for hospitalization for a simulated patient. This information included current inpatient medication orders, initial laboratory results, and current vital signs.” | 1 | “The game was arranged so that the students [the players] were required to take a stepwise approach to analyzing the case: assess the patient, determine a differential diagnosis, obtain objective results, make appropriate pharmacotherapeutic recommendations, and select acceptable monitoring plans.” “Questions to unlock additional clues included identification of drug-related problems, dose calculations, treatment selections, and monitoring parameters.” | 0 | Not explicitly reported | 1 | “The game was arranged so that the students [the players] were required to take a stepwise approach to analyzing the case: assess the patient, determine a differential diagnosis, obtain objective results, make appropriate pharmacotherapeutic recommendations, and select acceptable monitoring plans.” | 1 | “The game was arranged so that the students [the players] were required to take a stepwise approach to analyzing the case: assess the patient, determine a differential diagnosis, obtain objective results, make appropriate pharmacotherapeutic recommendations, and select acceptable monitoring plans.” | 1 | “The game was arranged so that the students [the players] were required to take a stepwise approach to analyzing the case: assess the patient, determine a differential diagnosis, obtain objective results, make appropriate pharmacotherapeutic recommendations, and select acceptable monitoring plans.” | 1 | Players received feedback for correctly unlocking boxes and solving clues | 0 | No instructor-led reflection was provided |
| **Kubin, 2020 (US)** | 1 | To begin the escape activity, students [the players] were divided into groups of six and were provided a bag containing an introduction to the scenario | 1 | “This escape activity progressed through three stages: assessment, diagnosis/planning, and intervention.” | 0 | Not explicitly reported | 1 | “This escape activity progressed through three stages: assessment, diagnosis/planning, and intervention.” | 1 | “This escape activity progressed through three stages: assessment, diagnosis/planning, and intervention.” | 1 | “This escape activity progressed through three stages: assessment, diagnosis/planning, and intervention.” | 1 | Players received in-game feedback from puzzles | 1 | “After the escape activity, a debriefing session was conducted to clarify key concepts, answer student [the players] questions, and recap the scenario.” |
| **Luu et al., 2020 (US)** | 1 | Players had access to patient portfolio's | 1 | ‘The user [the players] is then able to type in orders including laboratory tests, imaging studies, and procedures.” | 0 | Poorly described | 0 | Poorly described | 0 | Not explicitly reported | 1 | Appropriate intervention ordered | 1 | Players received feedback through patient deterioration | 0 | No instructor-led reflection was provided |
| **Middeke et al., 2018 (Germany)** | 1 | “All students [the players] were presented with four unknown cases in EMERGE.” | 1 | “Players take on the role of the attending physician and are prompted to take medical histories, choose appropriate diagnostic tests, identify the most likely diagnoses and take adequate therapeutic measures while treating up to ten patients simultaneously.” | 0 | Not explicitly reported | 1 | “Players take on the role of the attending physician and are prompted to take medical histories, choose appropriate diagnostic tests, identify the most likely diagnoses and take adequate therapeutic measures while treating up to ten patients simultaneously.” | 0 | Not explicitly reported | 1 | “Players take on the role of the attending physician and are prompted to take medical histories, choose appropriate diagnostic tests, identify the most likely diagnoses and take adequate therapeutic measures while treating up to ten patients simultaneously.” | 1 | “After having transferred a patient to a specific care unit, students [the players] receive a digital feedback on their diagnosis and treatment.” | 0 | No instructor-led reflection was provided |
| **Middeke et al., 2020 (Germany)** | 1 | “Upon a patient's arrival in the virtual emergency department, a pager message displays with brief information on the patient's name, sex, and main symptom.” | 1 | “Subsequently, game tasks include the following: taking a history by choosing from an alphabetical list of 70 preformulated questions, ordering appropriate diagnostic and laboratory tests, identifying the most likely diagnosis by choosing from a list of more than 130 differential diagnoses, taking adequate therapeutic measures, and transferring the patient to the corresponding care unit.” | 1 | “First, the student [the player] needs to assess the urgency of the situation and to consider all the relevant differential diagnoses.” | 1 | “Subsequently, game tasks include the following: taking a history by choosing from an alphabetical list of 70 preformulated questions, ordering appropriate diagnostic and laboratory tests, identifying the most likely diagnosis by choosing from a list of more than 130 differential diagnoses, taking adequate therapeutic measures, and transferring the patient to the corresponding care unit.” | 0 | Not explicitly reported | 1 | “Subsequently, game tasks include the following: taking a history by choosing from an alphabetical list of 70 preformulated questions, ordering appropriate diagnostic and laboratory tests, identifying the most likely diagnosis by choosing from a list of more than 130 differential diagnoses, taking adequate therapeutic measures, and transferring the patient to the corresponding care unit.” | 1 | “As the patient immediately responds to all medical treatments and interventional procedures, the consequences of students' [the players] decisions and actions can be experienced directly during the gaming session.”  Also, players received digital feedback and recommendation from a senior physician | 0 | No instructor-led reflection was provided |
| **Pisano et al., 2020 (US)** | 1 | “Each team initially picked a card that had a number corresponding to a hypothetical patient with anemia of unknown cause.” | 1 | “If the team answered the question correctly, it earned money enabling it to purchase diagnostic information.” | 1 | “The diagnostic workup phase (optional)” | 1 | ‘At the end of the 3-minute turn, teams could optionally choose to provide their anemic patient with a presumptive diagnosis. If the correct diagnosis was established, the team was given a new patient at the start of its next turn.” | 0 | Not explicitly reported | 1 | Optimizing test selection by considering information gleaned versus test cost | 1 | Upon getting results of a test, learners get real time feedback into the value-based care of that test | 0 | No instructor-led reflection was provided |
| **Raupach et al., 2021 (Germany)** | 1 | “Virtual patient cases presented in weeks 2 and 6 were similar albeit not identical with different ages, symptoms and observations to avoid simple recognition” | 1 | “In the game, students [the players] take on the role of the attending physician and need to triage virtual patients as well as take a history, order laboratory and other diagnostic tests make a diagnosis, initiate treatment, and transfer patients to the most appropriate care unit within the virtual hospital.” | 0 | Not explicitly reported | 1 | “In the game, students [the players] take on the role of the attending physician and need to triage virtual patients as well as take a history, order laboratory and other diagnostic tests make a diagnosis, initiate treatment, and transfer patients to the most appropriate care unit within the virtual hospital.” | 0 | Not explicitly reported | 1 | “In the game, students [the players] take on the role of the attending physician and need to triage virtual patients as well as take a history, order laboratory and other diagnostic tests make a diagnosis, initiate treatment, and transfer patients to the most appropriate care unit within the virtual hospital.” | 1 | Players received digital feedback and recommendation from a senior physician | 0 | No instructor-led reflection was provided. In-game answering questions by senior physician |
| **Smith and Davis, 2021 (US)** | 1 | “Questions provided brief clinical scenarios and required students [the players] to make safe decisions about medications.” | 0 | Not explicitly reported | 0 | Not explicitly reported | 0 | Not explicitly reported | 0 | Not explicitly reported | 1 | “Prioritize assessments and nursing interventions for patients receiving pharmacological therapies.” “Choose appropriate nursing actions and clinical decisions to complete the escape room at 100%.” | 1 | “After the students [the players] submitted their results, they were able to review their questions and their answers.” | 0 | No instructor-led reflection was provided |
| **Smith and Paul, 2021 (US)** | 1 | Players were presented with three cases | 1 | Identifying signs and symptoms (found in supplemental digital content) | 1 | Identification/rationale essential items (found in the supplemental digital content of the scientific article) | 1 | “Teams completed a nursing care plan for each Escape Room, developing/prioritizing nursing diagnoses and creating specific nursing interventions.” | 1 | “Teams completed a nursing care plan for each Escape Room, developing/prioritizing nursing diagnoses and creating specific nursing interventions.”  “Additional activities requiring nursing judgment, preparing for anticipated patient treatment, and skill performance were also created.” | 1 | “Additional activities requiring nursing judgment, preparing for anticipated patient treatment, and skill performance were also created.” | 1 | Feedback on puzzles and tasks | 0 | No instructor-led reflection was provided |
| **Sullivan et al., 2016 (US)** | 1 | Players were presented with multiple cases | 1 | “The cases are practicebased scenarios in which users [the players] can order laboratory tests, procedures, and imaging, just as they would in a real clinical setting.” “As part of the cases, students are required to make clinical decisions regarding what to ask in a history, what sort of patient examination to do, what laboratory and radiology tests to order, and both nonsurgical and surgical treatments.” | 1 | “The VSPC software tracks choices that students [the players] make in terms of diagnosis and treatment and also assigns them either a positive or negative score (maximum 100) based on their decisions in the case.” | 0 | Text, images, and videos are used to present the cases to impart information that allows the student [the player] to make diagnostic and therapeutic decisions.”  “Students [the players] are given options to guide their decisions and differential diagnoses throughout the case.” | 0 | Not explicitly reported | 1 | Text, images, and videos are used to present the cases to impart information that allows the student [the player] to make diagnostic and therapeutic decisions.” | 1 | “Along with their scores, students [the players] are also given a breakdown of which decisions caused them to lose points in the case. In this way, the scores and feedback related to points deducted functioned as formative assessment.” | 0 | No instructor-led reflection was provided |
| **Tyo and McCurry, 2021 (US)** | 1 | “Each station included one set of clinical cue cards, which included information such as a lead-in scenario, a simulation patient photograph, assessment data, vital signs, and laboratory/diagnostic results.” | 0 | Not explicitly reported | 1 | “Case scenarios in the game include ambiguous clinical data designed to make the players think through the clinical reasoning cycle and process all of the information.” | 1 | “Once the group decided on a suspect [diagnose], they stopped the timer and an educator recorded whether they correctly identified the case on the scorecard.” | 0 | Not explicitly reported | 0 | Not explicitly reported | 0 | Not explicitly reported | 1 | “At the conclusion of the game, a structured debriefing was conducted for each station to solidify teaching points and clarify misinformation. The structured debriefing included guided reflection for each station.” |
| **Wu et al., 2021 (Taiwan)** | 0 | Not explicitly reported | 0 | Not explicitly reported | 0 | Not explicitly reported | 0 | Not explicitly reported | 0 | Not explicitly reported | 0 | Not explicitly reported | 1 | “During the game, a failure message appears when the answer is wrong, and an applause when the answer is correct. The game provides immediate feedback to the player and using a formative assessment method.” | 0 | No instructor-led reflection was provided |
| **Zehler and Musallam, 2021 (US)** | 1 | “The student [the player] reviews the written patient report, including prenatal care data, medical history, and labor and delivery summary” | 0 | Not explicitly reported | 1 | Underline/mark risk factors and quantify blood loss | 1 | Early or late onset PPH (postpartum hemorrhage) | 0 | Not explicitly reported | 1 | “A list of interventions is provided in the format of a word jumble. Players must unscramble and list all interventions and identify which are primary, secondary, or tertiary.” | 1 | Getting point for correct answers | 1 | “Following each activity, a debriefing occurred using the three phase technique of reaction, analysis, and summary.” |
| *Note: Each included game was independently reviewed by two reviewers (GK, MH), and any discrepancies were discussed and resolved by a third reviewer (TvH). A score of 1 indicates that the specific step of the clinical reasoning cycle* ^8^ *was explicitly reported in the game, while a score of 0 indicates that the step was not explicitly reported. Following each score, a rationale was provided to explain the basis for the assigned score.* | | | | | | | | | | | | | | | | |
